# The Influence of the Soaking Temperature Rotary Forging and Solution Heat Treatment on the Structural and Mechanical Behavior in Ni-Rich NiTi Alloy

**DOI:** 10.3390/ma15010063

**Published:** 2021-12-22

**Authors:** Patrícia Freitas Rodrigues, Rodolfo S. Teixeira, Naiara V. Le Sénéchal, Francisco Manuel Braz Fernandes, Andersan S. Paula

**Affiliations:** 1 Universidade de Coimbra, CEMMPRE, Department of Mechanical Engineering, R. Luís Reis Santos, 3030-790 Coimbra, Portugal; 2 Escola de Engenharia de Lorena da Universidade de São Paulo (EEL–USP), Materials Engineering Department (DEMAR), Lorena 12602-810, SP, Brazil; rodolfoteixeira@live.com; 3 Materials Engineering Section-SE-8, Instituto Militar de Engenharia—IME, Rio de Janeiro 22290-270, RJ, Brazil; naiaravls@gmail.com (N.V.L.S.); andersan@ime.eb.br (A.S.P.); 4 CENIMAT/I3N, Materials Science Department, NOVA School of Science and Technology, Universidade NOVA de Lisboa, 2829-516 Caparica, Portugal; fbf@fct.unl.pt

**Keywords:** shape-memory alloys, NiTi, thermomechanical process, synchrotron radiation, heat treatments

## Abstract

The structural and thermophysical characteristics of an Ni-rich NiTi alloy rod produced on a laboratory scale was studied. The soak temperature of the solution heat-treatment steps above 850 °C taking advantage of the precipitate dissolution to provide a matrix homogenization, but it takes many hours (24 to 48) when used without thermomechanical steps. Therefore, the suitable reheating to apply between the forging process steps is very important, because the product’s structural characteristics are dependent on the thermomechanical processing history, and the time required to expose the material to high temperatures during the processing is reduced. The structural characteristics were investigated after solution heat treatment at 900 °C and 950 °C for 120 min, and these heat treatments were compared with as-forged sample structural characteristics (one hot deformation step after 800 °C for a 30 min reheat stage). The phase-transformation temperatures were analyzed through differential scanning calorimetry (DSC), and the structural characterization was performed through synchrotron radiation-based X-ray diffraction (SR-XRD) at room temperature. It was observed that the solution heat treatment at 950 °C/120 min presents a lower martensitic reversion finish temperature (A_f_); the matrix was fully austenitic; and it had a hardness of about 226 HV. Thus, this condition is the most suitable for the reheating stages between the hot forging-process steps to be applied to this alloy to produce materials that can display a superelasticity effect, for applications such as crack sensors or orthodontic archwires.

## 1. Introduction

NiTi alloy is one the most-interesting shape-memory alloys (SMAs) due to its noticeable functional properties, namely, superelasticity and a shape memory effect, combined with excellent mechanical properties, high corrosion resistance, and biocompatibility [1].

Functional properties are originating from a reversible phase transformation between austenite (B2 cubic structure) and B19′ (monoclinic martensite) [1].

This transformation occurs as a temperature variation (TIM, thermally induced martensite) or by applying stress (SIM, stress-induced martensite) results, and it may take place directly from austenite (parent phase with B2 cubic symmetry; space group $(Pm\bar{3}m$) to martensite (product phase with B19′ monoclinic symmetry; space group $(P2_{1}/m$), or it may go through an intermediate R-phase (trigonal symmetry; space group $(P\bar{3}$). The superelasticity occurs by the stress-induced martensitic transformation (SIM) [1,2].

These functional properties are guaranteed by the combination of the chemical and microstructural homogeneity. These characteristics are acquired through the most-suitable thermo-mechanical processing route and heat treatment [3].

Based on the functional properties, the precise composition control through thermomechanical and/or thermal processing (from melting processing to aging heat treatment) is the most important challenge [4,5,6].

NiTi alloys are reactive at the melting temperature, e.g., the titanium can form oxides, and carbides, due to their strong reactivity. To avoid these contaminations, the melting of these alloys should be carried out either in a vacuum or an inert atmosphere.

The hot working applied during the thermomechanical processing is important to set up the homogenization of the microstructure by the reduction in the solidification grain texture, and when a suitable reheating temperature is adopted, it is more effective than a simple annealing treatment for homogenization with 24 to 48 h. The literature indicates that this process is usually performed at a temperature range from approximately 800 °C to 950 °C [1], but the material needs processing in a temperature of at least 800 °C to function in such a way as to significantly decrease critical stress for the dynamic recrystallization to take place [7,8,9]. However, there are few works in the literature that detail the procedures to adopt rotary forging for thermomechanical-process geometries in the bar and wire forms of the NiTi alloys [4,8,10]. Rodrigues [11] highlights in his study thermomechanical processing via multi-step rotary forging, which uses some intermediate heat treatments in order to reheat to a suitable temperature for hot mechanical work, i.e., before each deformation step, a high austenite plasticity is required.

Usually, studies show heat treatment and a hot deformation temperature with a range of temperatures from 700 °C to 1000 °C for different treatment times [4,6,11,12,13]. Nevertheless, there is a gap in research that needs to be filled for solution heat treatments applied between rotary forging steps at 900 °C and 950 °C with a soaking time of 120 min, for forged NiTi alloys’ behavior.

Yeung et al. explored the effects of the heat-treatment process in the austenite phase transition temperature on a near-equiatomic NiTi alloy and adjusted the transition temperature by heat treatment. This study applied heat treatment at 800 °C, 850 °C, and 900 °C for 1 h. They reported that the austenite transition can be manipulated by adjusting some heat-treatment parameters such as the time and the temperature of the heat treatment. However, the heat treatment is the most-critical factor to change the transition temperature [4].

Paryab et al. studied the effect of different heat-treatment parameters on the microstructure and the hardness of NiTi (58.5 at.% Ni) SMA. They studied the heat treatment at high temperatures above 800 °C. This results in a higher M_s_ temperature as compared to the matrix at 700 °C [12].

The time of 120 min of heat treatment was used based on different studies that used this heat treatment to obtain a homogenized matrix. A. Safdel et al. [14] and S. Jiang et al. [15] subjected the NiTi sample with a nominal composition of Ni50.5 (at.%) and Ni50.9Ti49.1 (at.%), respectively, to a solution treatment held for 120 min at 850 °C and then water quenched the material.

In the previous thermomechanical processing scheme, all the intermediate annealing steps before each hot/cold forging were performed at 800 °C for 30 min, before the first hot deformation step, and 10 min, before the other hot and cold deformation steps, but with a crack’s significant incidence during processing [11]. In this study, it is proposed to analyze the effect of a soaking time of 120 min with higher temperatures (900 °C and 950 °C) for the solution heat treatment as compared with first step of hot forging with previous reheating at 800 °C for 30 min. This was done to observe if this time and temperature are enough to ensure no influence of impurities and a precipitate-free matrix, thus favoring the occurrence of B2-austenite at room temperature.

The influence of these heat treatments was analyzed by several techniques: ultra-microhardness, differential scanning calorimetry (DSC), optical microscopy (OM), and synchrotron X-ray diffraction (SR-XRD). The structural study by the SR-XRD technique is a reliable precise technique, mainly because it allows the identification of the lower size precipitates (Ni_3_Ti and Ni_4_Ti_3_) and the observation of a greater sample area with a reduced spot size while keeping an intense beam. The beam can be precisely directed to the different regions along the sample diameter. Besides, its energy is considerably higher (up to 100 keV) than laboratory sources (low-energy X-ray). The use of this technique allows for greater penetration in the analyzed materials. Even when using thick samples, it is possible to work in transmission mode.

The SR-XRD combined with DSC techniques allows checking the structural state of the materials and its transformations characteristics. The SR-XRD results give a precise identification of the phases that are present at room temperature and allows to understand the material’s behavior after heat treatments or just the first step of the hot forging applied. While the DSC technique gives a precise definition of the transformation temperature ranges, it does not always provide clear identification of the individual phase transformations due to partial overlapping of transformation peaks. Besides, the ultra-micro-hardness was applied to investigate the mechanical properties, especially the super-elastic behavior. These methods were applied to determine the effects of the heat treatment on the transformation temperatures, the structure, and the mechanical properties, which are very important properties for the design/manufacturing of the wires for the orthodontic application.

Therefore, the main objective of the present work is, for the first time, to attempt to substantially optimize the reheating stage applied before each step of the rotary-forging process steps based on the results of the microstructural and mechanical behavior on the solution heat treatment. This was done to ensure the suitable temperature for processing the material, taking into account the transport of the material from the furnace to the forge. The study of these heat treatments can be considered as an important stage to optimize the homogenization process and as a reheating stage to apply the hot deformation at a suitable temperature to produce shape-memory alloys.

## 2. Material and Methods

### 2.1. Material

**Figure 1:**
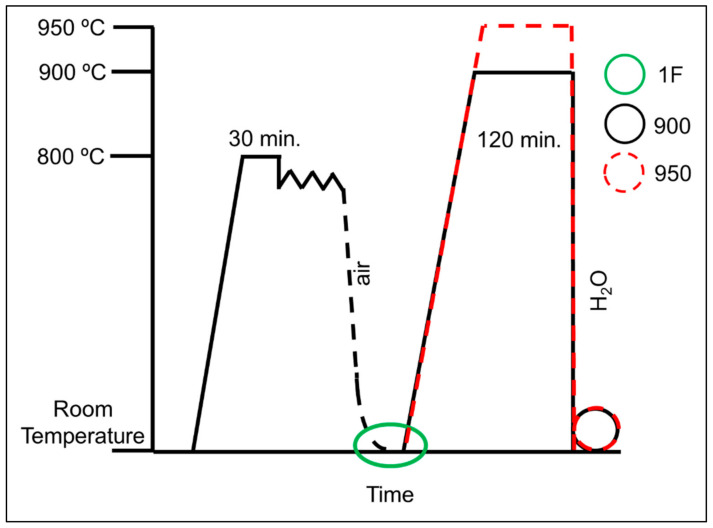
Scheme of the thermomechanical processing and subsequent solution heat treatment explored in this study.

An Ni-rich NiTi alloy ingot was produced by a vacuum-induction-melting (VIM) furnace under an argon atmosphere using a graphite crucible was used; the base material had a nominal composition of 50.8 at.% Ni. The small part of the VIM ingot was remelted by vacuum arc remelting (VAR). In the current forging process, the remelted ingot was submitted to hot forging (1F sample), which consisted in previous reheating with soaking at 800 °C for 30 min, in a muffle furnace before transfer to a four-hammers tool (10.41 mm diameter), which was followed by hot deformation and slow cooling to room temperature. Hot forged samples were cut off (1 mm length and 10 mm diameter) and were submitted to solution treatment at distinct temperatures, 900 °C (900 sample) and 950 °C (950 sample), both for 120 min followed by quenching into the water. The heat treatment for all the NiTi samples was carried out in a conventional furnace similar to what was used on the reheat processing for hot forging. Figure 1 summarizes the thermomechanical and subsequent heat-treatment processes explored in the present work.

**Figure 2:**
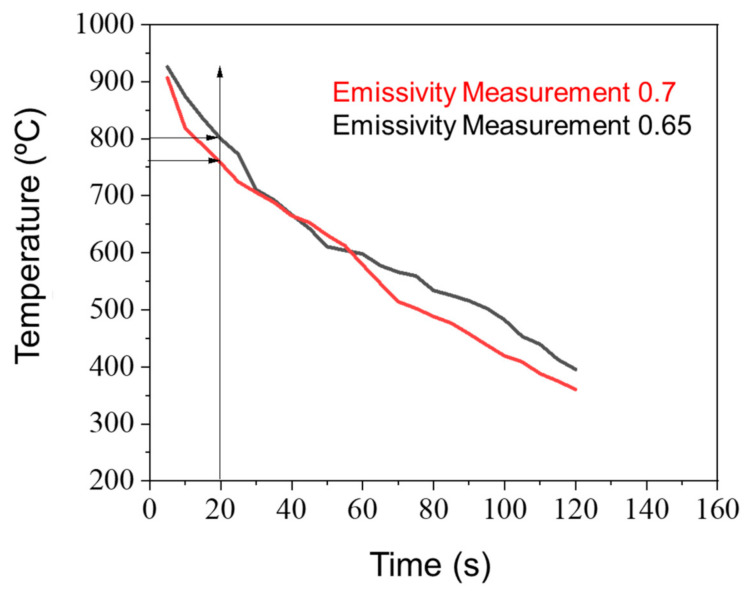
Emissivity measurement (0.65 and 0.7) of air-cooling curve of the NiTi alloy samples after soaking on the heat treatment at 950 °C.

In addition, the monitoring of the cooling of the material reheated to 950 °C, with an optical pyrometer configured with emissivity values of 0.70 and 0.65, was performed. The curve obtained is a function of the time between the removal of the material from the furnace to the insertion in the forge to deformation of the material (Figure 2), estimated in the time interval of 15 and 20 s, in the hypothetical case of applying 950 °C instead of 800 °C in the previous reheat for hot deformation used in this study. These cooling curves allow us to state that the material was heat-deformed at a temperature below 800 °C when reheated to 800 °C.

### 2.2. Microstructural and Mechanical Characterization

The microstructure of the NiTi alloy was examined at room temperature in the as-forged condition and after the solution treatments (900 °C/120 min and 950 °C/120 min). Samples for optical microscopy (OM) were mounted, polished, and etched using a solution of 15% HF, 45% HNO_3_, and 40% H_2_O (by volume) to reveal the grain size. For the determination of the average grain size, the intercept procedure was applied following ASTM E-112-96 [16].

The characterization of the transformation temperatures was performed by differential scanning calorimetry (DSC) and the structural characterizations by synchrotron X-ray diffraction. For the DSC analysis, samples were analyzed in a DSC 204 F1 Phoenix (NETZSCH-Gerätebau GmbH, Wittelsbacher straße, Germany), with thermal cycles from −150 to 150 °C and a heating/cooling rate of 10 K·min^−1^.

For the XRD analysis, synchrotron radiation XRD (SR-XRD) was used. The SR-XRD experiment was performed in transmission mode using a disc-shaped sample (10 mm diameter and 1 mm thick for all samples) and at room temperature (around 25 °C, without a controlled room temperature). SR-XRD was performed at beamline P07 High-Energy Materials Science (HEMS) of Petra III/DESY, using a wavelength of 0.1426 Å (87 keV), and a two-dimensional (2D) detector PERKIN ELMER XRD 1621 was placed at 1.35 m from the sample. The raw 2D images were treated using the Fit2D program [17] to calculate the individual XRD patterns by integration from 0° to 360° (azimuthal angles). For the SR-XRD measurement, the identification of the diffraction peaks was performed based on the ICDD database.

The instrumented ultra-micro hardness analysis was performed by a Vickers indenter (Shimadzu brand, model DUH-211S) using a maximum load of 20 gf/196.133 mN, with a load speed of 13.3240 mN/s and a hold time at load/unload of 20 s. In each sample, five tests were performed along the ½ thickness. All analyses were performed at room temperature (with the controlled temperature at 20 °C) in order to observe evidence of the super-elastic characteristic that may have been developed in these three structural conditions under study (1F and heat treated at 900 and 950 °C).

## 3. Results and Discussion

### 3.1. Structural Characterization

**Figure 3:**
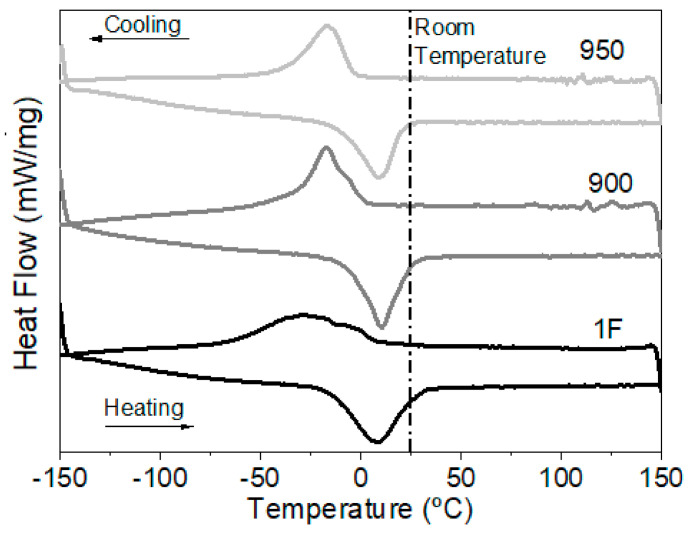
DSC plots of 1F, 900, and 950 samples.

Figure 3 shows the DSC plots of the 1F sample, heat-treated sample at 900 °C for 120 min (900 °C sample) and heat-treated sample at 950 °C for 120 min (950 sample). Multiple peaks superimposed were observed for 1F sample DSC results, which were attributed to the previous deformation imposed and/or the compositional inhomogeneity of particles [18,19,20], while the heat-treated samples showed the single-phase transformation peak, on cooling as well on heating. The temperature hysteresis of the 900 and the 950 samples was similar, but the transformation temperatures were different.

Table 1 shows the phase-transformation temperatures’ evolution with the heat-treatment temperature compared to the forged condition. From the DSC measurements, it is visible that the transformation temperatures (namely, A_f_ and M_s_) had slightly shifted to lower temperatures with solution heat treatment (900 and 950 °C).

When comparing the 1F sample with the 900 sample, M_s_ and M_f_ were decreased by 7 °C and 33 °C, respectively, while A_s_ and A_f_ were decreased by about 11 °C and 4 °C, respectively. These changes can be attributed to the solution heat treatment that may hinder the martensite nucleation, promoting residual stress relief as well as increases in the Ni matrix concentration and, consequently, decreases in the transformation temperature [21].

When comparing the 900 sample with the 950 sample, M_s_ and M_f_ were decreased by 6 °C and 4 °C, respectively, while A_s_ and A_f_ were decreased by about 5 °C and 3 °C, respectively. This behavior is similar to a comparison between the 1F and the 900 samples.

**Figure 4:**
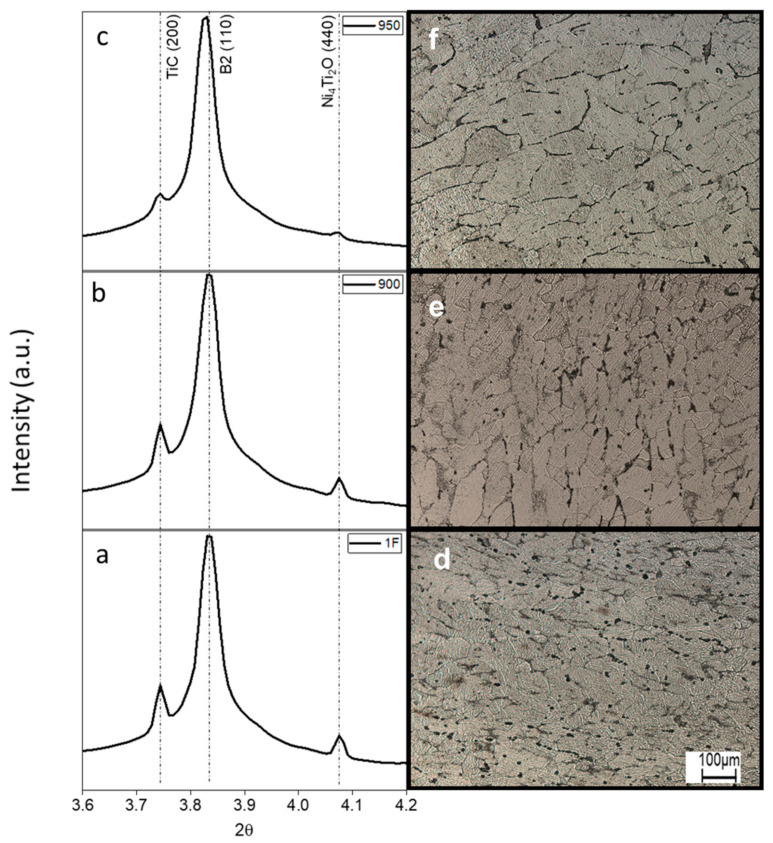
XRD diffractograms and microstructure at as-forged (**a**,**d**) and heat-treated at 900 (**b**,**e**) and 950 (**c**,**f**) samples at room temperature.

Diffractograms of the 0 to 360° integration of the Debye–Scherrer rings are presented in Figure 4a–c. Indexing of the diffraction patterns shows that, for all the samples, the Ti_4_Ni_2_O and TiC are present as well as for the 1F sample (Figure 4a–c). All samples were fully austenitic without evidence of any precipitate (Figure 4a–c), and even the A_f_ temperature of the 1F sample was above room temperature.

The optical micrographs (Figure 4d–f) show the difference in the grain size. Grain size increased when the temperature of the heat treatment increased, as shown in Table 2. The 950 sample evidenced grain growth, as supposed in the SR-XRD results by larger d-spacing values.

Thus, the solution heat treatments at 900 °C/120 min and 950 °C/120 min guarantee a fully austenitic matrix but promote grain growth, as expected by the high temperatures, applied without a mechanical component, which increase the microstructural modifications aided by the diffusion process.

The fully austenitic matrix was observed in the hardness results, as shown in Table 2. The high Vickers hardness value was observed in the 1F sample (302.97 HV). The DSC result shows the A_f_ temperature at 27.8 °C, and the hardness measurement was performed at 20 °C with the temperature controlled, which probably promoted B2 and R-phase mixture observations on the matrix. B2 → R-phase is the transformation that can promote internal stresses and reveal a high hardness [22].

There were differences in hardness from the 1F, 900, and 950 sample surfaces. This implies that strain recovery and recrystallization were accomplished by solution treatment at 900 °C and 950 °C for 120 min. Besides, it suggests that there is the homogeneous distribution of internal stresses remaining in the heat-treated material and/or the grain growth contributing to the decrease in the Vickers hardness value. Additionally, it is important to note that as the hardness changed with heat treatment temperature, the phases present at the measurement temperature performed also changed, since the transformation temperature of R-phase and B2 austenite varied with the increasing heat-treatment temperature [22].

**Figure 5:**
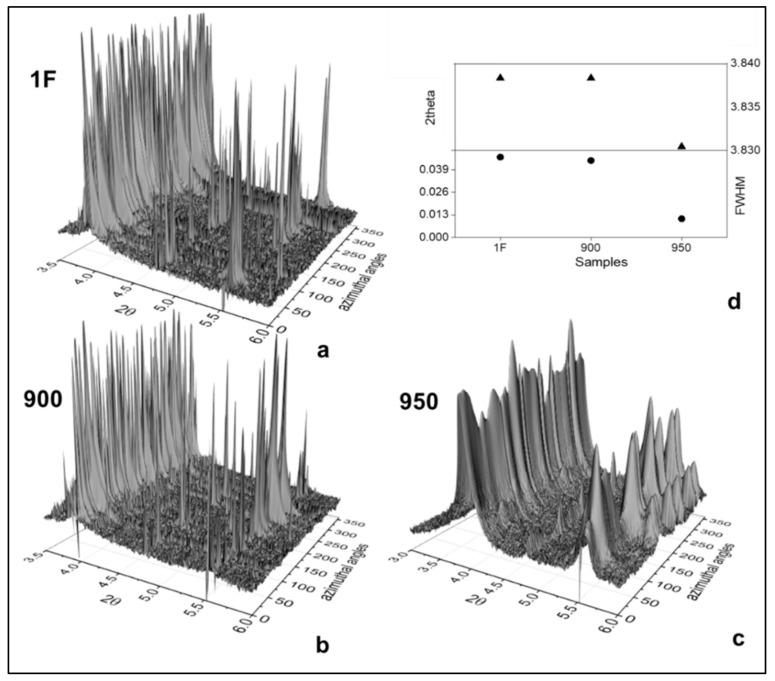
3D plots transform of Debye–Scherrer diffraction rings (azimuthal angles) vs. 2θ for (**a**) as-forged (1F), (**b**) 900, and (**c**) 950 samples and (**d**) 2θ and FWHM evolution.

The grain growth can also be observed in Figure 5a–c, where the intensity versus 2θ is plotted as a function of the azimuthal angle (ϕ). The azimuthal angles show many discontinuities and isolated peaks of very high intensities. Figure 5d shows the B2(110) FWHM and 2θ analysis. A decreasing behavior could be observed for both, indicating the possibility of the internal compression stress, as a consequence of an increase in d-spacing due to grain growth.

### 3.2. Mechanical Behavior

**Figure 6:**
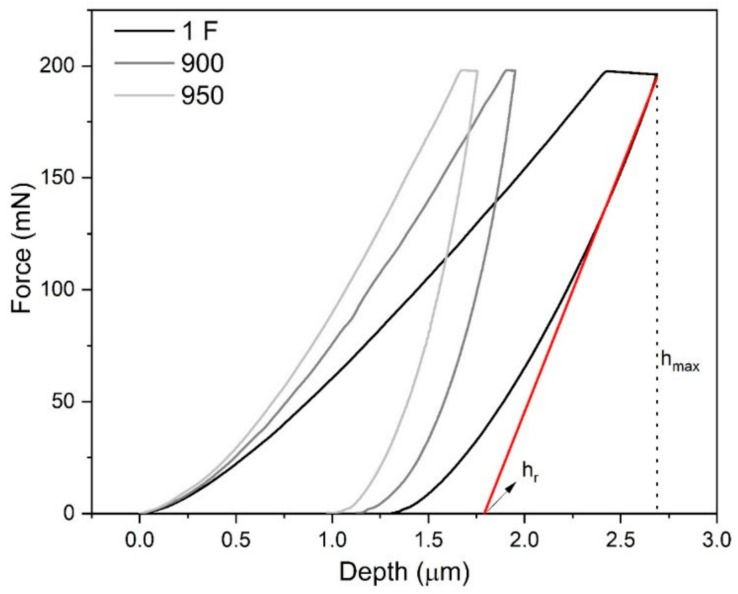
Force vs. depth curves (loading and unloading).

The behavior of curves during the loading/unloading showed the effects between the temperature of heat treatment and the forged sample (Figure 6). When the temperature of heat treatment increases, the maximum depth (h_max_) increases, and the same behavior occurs with the as-forged (1F sample) condition. It is possible to calculate h_r_, which is derived from the force-displacement curve and is the intercept of the tangent to the unloading curve. The smaller values of h_r_ represent a more elastic return, and better results were observed for the sample treated at 900 °C for 120 min. The as-forged sample exhibited a plateau region, which could have been associated with the stress-induced martensitic transformation. DSC analysis observed the presence of the R-phase, at the measurement temperature (20 °C), which would justify this conclusion. The same behavior was identified in the study Chiang et al. It is emphasized that the presence of mix phases (B2 and R-phase) increased the hardness [22].

**Figure 7:**
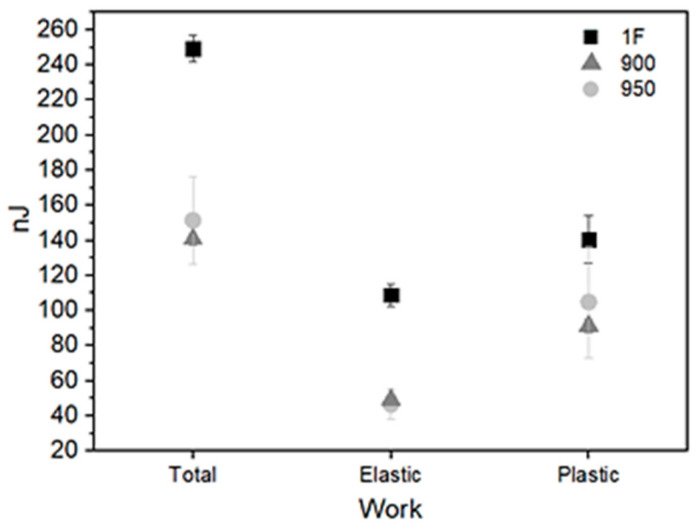
Total, elastic, and plastic work.

The total and elastic work were calculated using the areas below the loading and unloading curves, respectively, as shown in Figure 7. When comparing the solution heat-treatment temperatures, the elastic work was higher than 900 °C; however, the error bar for 950 °C was also high and showed values higher and smaller than 900 °C. The same behavior was observed in total and plastic works, where the plastics bar error for the temperature of 950 °C was close to the 1F sample. The higher error for the temperature of 950 °C might be justified for the high growth of grains. The 1F sample shows higher values in all works; however, the presence of the R-phase at the measurement temperature was observed, and the A_f_ is above the influence in the properties.

The hot forged sample was annealed before the forging steps, assuring the sample was in an austenitic field. Due to the loss of temperature between the forging reduction steps, the sample should be reheated at 950 °C for 10 min in order to assure that the thermomechanical process occurs at about 800 °C. The curve obtained is a function of the time between the removal of the ingot from the oven to insertion in the forge and deformation of the material, which was estimated in the time interval of 15 and 20 s (Figure 2). With the emissivity of 0.70 and a time of 20 s, the ingot presented a temperature of 760 °C, and with an emissivity of 0.65 for 20 s, it presented a temperature of 800 °C.

The verified temperature at the end of the time corresponding to the material transport time presented a value close to the temperature used in the current processing (750 and 800 °C), with ideal values to the processing of this type of material. However, as the material in the current thermomechanical processing (1F) was reheated at a temperature of 800 °C, the processing temperature for previous reheating to rotary forging was certainly lower than the ideal temperature for processing this material. This affirmation is supported by the literature, which reported that this deformation temperature must be adequate for deformation of this material, since it does not promote the significant oxide layers’ formation and since it facilitates material deformation by the occurrence of dynamic recrystallization [1]. However, it is important to emphasize that, with the reduction in the cross section of the bar with the progress of the reduction steps in forging, the loss of temperature in the transport of the reheating-step interpass from furnace to the forge becomes more significant.

## 4. Conclusions

Ni-rich NiTi alloys forged by one hot step and subsequent heat-solution treatment were investigated by using DSC, SR-XRD, ultra-micro-hardness, and optical microscopy techniques. The results obtained can be summarized as follows: 900 °C/120 min and 950 °C/120 min samples showed an A_f_ near to room temperature. All these conditions assure that the hot forging process will occur in the austenitic field.

Therefore, in the SR-XRD and DSC investigations, it is visible that, for the solution heat treatment between forging steps, the temperature of 950 °C was indicated.

Firstly, this was the case because the lower phase-transformation temperature assures the stability of the austenite phase at room temperature. Secondly, in this condition, the matrix is fully austenitic and is suitable to apply the thermomechanical process proposed. Thirdly, the super-elastic behavior observed in the ultra-micro-hardness curves supported the functional property presence because of the first forging-step thermomechanical process. Finally, since the indicated hot processing temperature for these alloys was about 750–850 °C, the preliminary reheating step at 950 °C can assure that forging will be carried out on about 800 °C, taking into account the transport of the material from the furnace to the forge, in order to ensure the occurrence of dynamic recrystallization to provide microstructural homogenization by high hot plasticity of the NiTi alloys.

## Figures and Tables

**Table 1:** Phase-transformation temperatures.

	Cooling (°C)		Heating (°C)	
	M_s_	M_f_	A_s_	A_f_
1F	8.1	−3.9	−15.1	27.8
900	1.2	−30.2	−4.4	23.6
950	−5.1	−36.4	−9.0	20.7

**Table 2:** Average values of the grain size by ASTM E-112-96 [11] (µm), and the Vickers hardness (HV) values.

Sample	µm	HV
1F	58	302.97
900	61	252.77
950	93	225.98

## Data Availability

Not applicable.
